# Remarks on Dr. Kinglake's Opinions Concerning the Obstetric Art

**Published:** 1816-05

**Authors:** John Wayte

**Affiliations:** Calne.


*For the London Medical and Physical Journal.*

### *Remarks on Dr. Kinglake's Opinions concerning the Obstetric Art;* by JOHN WAYTE, Esq. of Calne.

YOUR Journal for March, through some mistake in the bookseller's agent, has been detained, and is now arrived with that for April. Dr. Kinglake appears firmly wedded to his own opinions on obstetric practice, and has replied at great length to Mr. Atkinson and myself; and, although our views and reasonings upon the subject have had no influence whatever, I should suppose that Dr. Merriman's reply will carry complete conviction. Most probably (but for the above-mentioned delay) Dr. Kinglake would have received a few lines from me earlier, for, in justice to myself and the profession, I could not have abandoned the cause, until convinced of the soundness and superiority of his proposition. Dr. Merriman has observed that his first letter might have been consigned to oblivion without comment;—but to sit tacitly and read such undeserved revilings against the art, and unhandsome reproaches against its practitioners, was more than my feelings could allow. The cause has been so ably advocated by other hands, I need not trespass much upon your pages.

In the second paragraph of Dr. Kinglake's reply, he makes makes me "gravely affirm that Nature's provision for parturition is too insufficient to be safely left to its own resources," and appears to him to be "far-fetched:" I say it is very far-fetched; but it is the Doctor's own fetching, as I never affirmed Nature's insufficiency *in toto*, nor proclaimed her all-sufficiency. My expression was—"without the least irreverence, or without detracting from the powers and perfections of Nature, I do suppose and know that she is inadequate to accomplish *every* requisite at *all* times, respecting the birth of the human species:"—had, then, the word *occasional* been added to *insufficiency*, my meaning would not have been perverted. In the latter part of the same paragraph there is some obscurity of language (at least far from sound reasoning) when he says "The accidental insufficiencies of Nature are not her original handy-work: they are, in *every* instance, morbid deviations from the primitive perfection under which she invariably appears." Can he believe primitive perfection invariable, whilst so many unfortunate *lusus naturæ* are seen? or, when calling all deviating insufficiencies morbid, does he mean to compare a distorted pelvis from ruchitis, which is morbid, with an arm-presentation, which is not morbid? Yet, each proves Nature's insufficiency during parturition. It is admitted, likewise, that "Nature's works are equal to all their ends; and that it is with the deviations from the natural standard of perfection that the hand of art has to do." Now, how shall we find that exactly possible? to call Nature unerring, and equal to all their ends, yet with deviations, and requiring the hand of art;—thus establishing as a corollary that Nature, in so deviating, always requires watching, and occasionally assistance. If my argument, gentlemen, evinces any misconception of the structure of those sentences, I remain open to your correction.

In the ensuing paragraph, where attempts are made to refute my argument with respect to placental presentations, I am astonished at the different epithets given to Nature, which, after being so much extolled as to be "universally perfect," and her works equal to all their ends, is now not only termed deviating (properly), but suffering "an inversion," taking a "topsy-turvy course not reasonably to be calculated on," and, I hope, after this concession, not infallibly depended on. I will not call the correctness of that information in question, respecting the proportionate number of practitioners that have met with this species of presentation, although it does not coincide with another statement: I will, however, mention, that there are only four practitioners in this town, yet each has met the case, and two two of them several times. To myself (who have been in practice five years) it has occurred; and, as "it is not clear that a placental presentation, unrelieved and unperforated for the manual delivery of the fœtus, would terminate in death," I will, with all deference, submit it to Dr. Kinglake's perusal. I was sent for in great haste to attend a woman, at the distance of three miles, in labour with her first child, whom I found on the bed flooded to an alarming degree. Her mother informed me that it commenced about an hour prior to my arrival, together with very trifling pains. I apprehended it to be a placenta case, which, on examination, was not ill-founded. Clear as to its nature and danger, satisfied also of the steps necessary to be taken, I informed her relations of every circumstance as to treatment and probable result. The os uteri was not dilated more than to the extent of half-a-crown; the placenta plainly to be felt; and the flow of blood continual. Prior to any hasty determination on perforating its body, I passed a portion of my finger round within the os uteri, in order to ascertain whether its thin edge was within reach, so that the membranes might be broken. Fortunately I found it towards the os pubis, and immediately ruptured them. Hæmorrhage diminished, and the pains grew somewhat stronger. The presentation was natural; but, from the circumstances of its being a first labour, an undilated os uteri, with great prostration of strength, (her pulse being scarcely perceptible at the elbow) I could not allow delivery to be natural, or else death would have come natural. And now I will make an appeal to the whole medical public, to say whether I was not warranted in gradually dilating the parts, and effecting delivery by the feet? or whether the uterine contraction, after Dr. Kinglake's mode of reasoning, would have been preferable? My patient, I am happy to add, recovered with an opiate draught.

Once more I must assert the occasional necessity (it matters not how seldom) of assisting the dilatation of a rigid os uteri, on these grounds:—The membranes, with their fluid contents in due quantity, are known to be the natural, most easy, and effectual means of dilating the soft passages, preparatory to expulsion, by being gradually protruded and collecting exteriorly to the os uteri. It is well known that cases are occasionally offering themselves where the quantity of waters is very small (not exceeding one table-spoonful), the os uteri very unyielding throughout forty-eight hours' regular pains, causing the powers of nature to flag, indicating much distress in accomplishing her ends, and plainly asking for proper co-operation from art. Not,

 therefore, therefore, until undue interference is made evident, and the practice indiscriminately followed, can the terms "intermeddling or mischievous" become applicable.

Neither, until some further light is thrown upon the subject, shall I cease to render proper aid in face presentations, which are now (for the first time) ranked among "slight deviations from the right line of exclusion," and "rather the momentary effects of the contractile action on the presenting part, than a stationary position." From which reasoning one would be led to suppose that face cases were never laborious, never required instrumental aid; but that they would as readily become favourable by the same "contractile action" as made them unfavourable.

Profuse uterine hæmorrhage, which, with convulsions, have been usually deemed the most dangerous occurrences in midwifery, seems to excite no great apprehensions of alarm in Dr. Kinglake's mind, as he would fain have us leave such "afflicting occurrences" to Nature and syncope,—not recollecting that fatal syncope has been known to occur from this cause. Entertaining these notions, only suppose the case that your patient happened to die whilst you were seated before the fire instead of at her bed-side, and you were questioned as to the treatment pursued;—the reply must be that you advised a "low temperature and the recumbent posture." Both undeniably proper. But did you not assist Nature before so much blood was lost as to cause syncope, by firm pressure on the uterus, particularly at its lower part, in order to promote its contraction? Did you not administer that well-known auxiliary a large dose of tinctura opii. And, if the symptom continued, did you not apply cold wetted cloths to the abdomen, and keep up a regular pressure until all danger was over? No. Then you certainly omitted measures calculated to ensure success, and secure your own reputation.

All I deem requisite to remark on my term *necessity* is to inform Dr. Kinglake "what sort" it is which caused midwifery to pass from the hands of women into those of men. Had midwives but possessed complete information in their calling, so as to have been found competent upon all occasions, and not shown the reverse by committing so many grand and irretrievable mistakes, there would have been no change, nor any *necessity* for it; but their ignorance becoming so palpable, created a *necessity* for better-informed practitioners, into a preference for whom females would not have been "cajoled" but for their known superiority in so important a concern.

With respect to the proportionate occurrences of preter-

 natural natural presentations, untoward labours, and the comparative successes of male and female practitioners, I shall advise a reference to the clear data of Dr. Merriman, for which I have to express myself under an obligation; and conclude this epistle with a few remarks on those unfounded charges against the whole body of male practitioners.

Dr. Kinglake says, that "were the ordinary practice of midwifery to be confined to women, much less would be heard of *preternatural* labours, *laborious* and *inefficient* efforts for natural parturition, and of resorting to manual and *instrumental* aid. Undisturbed nature would then proceed slowly, safely, and efficiently." "No *violence* would have been in any way added," &c. &c. &c. throughout the paragraph. Can such calumnious language be uttered decently by any man, much more by a searcher after philosophical truths? or can one sit stoically and read strong insinuations thrown out against gentlemen of creating preternatural labours; of increasing the sufferings of a fellow-creature, and the weaker sex, by adding "violence" to pain; and thereby dealing out misery, "disease," "mutilation," and "deformity"? But this is not the worst: he has really asserted that "the skilful, the instrumental accoucheur" has ("when no symptoms of imminent danger on the part of the mother have arisen") "bored the fœtal head," and taken away the life of a child unnecessarily!! That a person of Dr. Kinglake's acknowledged ability and eminent station should have advocated the cause he has, and especially with so much apparent rancour, must be a source of regret to scientific men, as it will most assuredly draw down censure and disgust from the whole medical world.

*April 13th,* 1816.

